# The solid state dispersion method for synthesizing eggshells ES/TiO_2_ composite to enhance photocatalytic degradation methylene blue

**DOI:** 10.1016/j.mex.2024.103150

**Published:** 2025-01-06

**Authors:** Rahmawati Munir, Dadan Hamdani, Darnah, Eva Marliana, Rahmiati Munir, Sahara Hamas Intifadhah, Ratna Kusuma

**Affiliations:** a Theoretical and Material Physics, Physics Department, FMIPA Mulawarman University, Samarinda, 75242, Indonesia; b Study Program of Statistics, Department of Mathematics, Faculty of Mathematics and Natural Sciences, Mulawarman University, Samarinda, 75242, Indonesia; c Department of Chemistry, Faculty of Mathematics and Natural Sciences, Mulawarman University, Samarinda, 75242, Indonesia; d Department of Physics, Faculty of Mathematics and Natural Sciences, Mulawarman University, Samarinda, 752429, Indonesia; e Department of Biology, Faculty of Mathematics and Natural Sciences, Mulawarman University, Samarinda, 75242, Indonesia

**Keywords:** SSD method, MB degradation, ES/TiO2 composite, Water purification, Photocatalysis, Solid State Dispersion (SSD)

## Abstract

The use of eggshells as a primary source for developing value-added materials has garnered significant attention in recent years due to their effectiveness as an excellent adsorbent and support. In this study, the Solid-State Dispersion (SSD) method was utilized to prepare composite photocatalysts of eggshells (ES)/TiO₂ in various ratios. TiO₂ and eggshell photocatalysts were also employed as control samples. The samples were characterized using X-ray Diffraction (XRD) to analyze the crystalline structure and phases, along with High-Resolution Transmission Electron Microscopy (HRTEM) to examine the morphology and surface structure at the microscopic level. These characterizations support the data analysis, offering insight into the relationship between structural and morphological properties and the photocatalytic performance of the composites. The photocatalytic efficiency of the composite was assessed in a suspension system using methylene blue (MB) solution as the target pollutant. Among the three different ratios, the ES/TiO2 (9:1) composite achieved the highest adsorption and 95.63 % photocatalytic degradation of MB. This indicates that the adsorptive capacity of eggshells is very high, even though it primarily serves as a support for TiO₂ in the ES/TiO₂ composite photocatalyst. The increased surface area of the TiO₂/eggshell composite photocatalyst enhanced MB solution adsorption and photocatalytic degradation, thereby improving its effectiveness. Overall, it can be concluded that eggshells have excellent potential as a support for photocatalysts and as an environmentally friendly catalyst. Some highlights in this article:

• The Solid-State Dispersion (SSD) method is presented as an efficient and eco-friendly approach for synthesizing ES/TiO₂ composites.
• ES/TiO₂ composites effectively degraded methylene blue (MB) by 95.63 % under UV light.
• This method promotes sustainability by reusing eggshell waste and improving environmental benefits through enhanced photocatalysis.

## Graphical abstract

**Figure:**
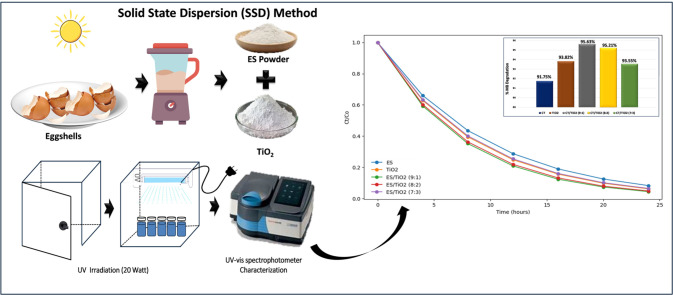

Specifications table

**Table:** 

Subject area:	Environmental Science
More specific subject area:	Wastewater Treatment
Name of your method:	Solid State Dispersion (SSD)
Name and reference of original method:	Olatabadi, S., Fattahi, M., & Nabati, M. (2021). Solid state dispersion and hydrothermal synthesis, characterization, and evaluations of TiO₂/ZnO nanostructures for degradation of Rhodamine B. Desalination and Water Treatment, 231, 425–435. https://doi.org/10.5004/dwt.2021.27496
Resource availability:	Laboratory equipment for synthesis and characterization, access to eggshells and TiO₂, analytical instruments for evaluating photocatalytic activity.).

## Background

In composite material synthesis, the Solid- State Dispersion (SSD) is a more economical and environmentally friendly method, as it only requires simple base materials and solvents like ethanol, reducing production costs and chemical waste [1]. This method also allows for better control over the distribution and composition of materials at the microscopic level, which is crucial for ensuring homogeneous integration of Titanium Dioxide (TiO₂) onto the Eggshells (ES) surface. With this control, the resulting composite exhibits good stability and optimal physical and chemical properties, making it suitable for application as a photocatalyst and adsorbent in water purification. These advantages make SSD the ideal choice for this study.

State Dispersion (SSD) method is selected due to its numerous advantages, making it a superior approach for developing ES and TiO₂-based composites. SSD involves mixing two or more solid materials, followed by heating to induce interactions between the materials without the use of complex solvents [2]. The main advantage of this method lies in its simplicity, as it does not require extreme conditions or specialized equipment such as autoclaves, making it easier to implement and more time-efficient.

The utilization of ES as an alternative solution in wastewater treatment has become an intriguing research topic in recent years [[3], [4]]. Eggshells are a bio-based source of calcium and present a renewable raw material with potential applications in material development industries [[5], [6], [7]]. The calcium carbonate (CaCO₃) contained in eggshells can strongly interact with heavy metals, facilitating the purification process of water contaminated with such pollutants [[7], [8], [9], [10], [11]]. Eggshells contain over 90 % CaCO₃ by weight, which has been used to prepare low-cost calcium oxide (CaO) catalysts [12]. The characteristics of eggshells as an adsorbent can be further optimized when composited with photocatalytic materials such as TiO₂.

TiO₂ possesses excellent photocatalytic activity, particularly under ultraviolet (UV) light. Its non-toxic nature, high oxidation behavior, stability, and low cost make it highly popular for various environmental applications. The anatase crystal phase of TiO₂ has proven to be particularly effective in producing electron-hole pairs. These electrons and holes trigger radical and ionic reactions with H₂O and O₂, which can decompose water and air pollutants [[13], [14], [15], [16], [17], [18], [19]]. Modified TiO₂ has shown great potential in the degradation of organic pollutants in water, offering promising solutions for addressing environmental water issues [20].

The objective of this research is to develop and optimize a composite material composed of ES and TiO₂ using the Solid-State Dispersion (SSD) method. The primary goal is to evaluate the composite's effectiveness in improving the photocatalytic degradation of organic pollutants, particularly methylene blue (MB), under UV light. This study also aims to analyze how different ES/TiO₂ ratios affect the photocatalytic performance, as well as the structural and morphological properties of the composite. Ultimately, this research seeks to contribute to the development of sustainable water purification technologies by utilizing biological waste materials, such as eggshells, in an environmentally friendly approach.

## Method details

### Materials

The raw materials used in this study were eggshells obtained from martabak egg traders around the Bengkuring housing, Samarinda city. Titanium Dioxide (TiO2), Ethanol (95 %), Aquades, Methylene Blue (MB) were purchased from the Samarinda Chemical Store, East Kalimantan.

### Preparation of eggshells (ES) powder

The preparation of ES powder involves a series of steps aimed at ensuring purity, uniformity, and optimal material characteristics suitable for research applications. Initially, cleaning the eggshells involves removing external contaminants and detaching the inner membrane, which are crucial for reducing organic residue and potential impurities that could interfere with material properties. Following this, immersion in hot water at 80 °C for 15 min sterilizes the shells, helping to reduce microbial presence and soften the structure, which aids in subsequent grinding. The preliminary drying process is then conducted by sun-drying the eggshells, effectively minimizing moisture content to enhance powder consistency.

**Fig. 1:**
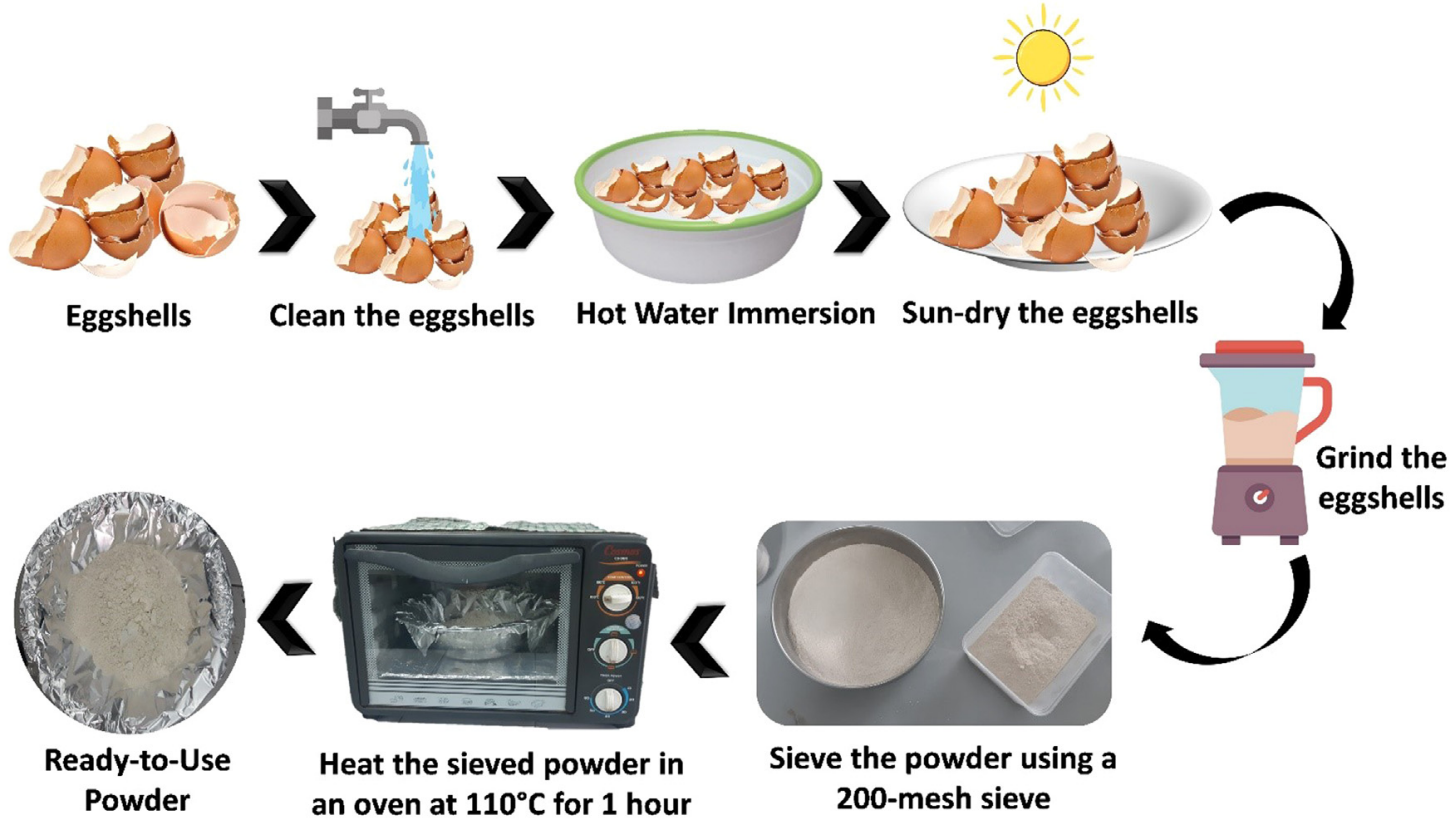
A schematic illustration of preparation of Eggshells (ES) Powder.

A schematic illustration of preparation of Eggshells (ES) Powder is shown in Fig. 1. Once dry, the grinding process is performed using a blender, which yields a coarse powder while retaining most of the material's structural integrity. This coarse powder is then subjected to sieving with a 200-mesh sieve to achieve a fine and uniform particle size distribution, ensuring optimal surface area and homogeneity necessary for consistent experimental results. Subsequently, the sieved powder undergoes thermal treatment in an oven at 110 °C for 1 h to eliminate any residual moisture, enhancing stability and storage potential. After heating, the powder is cooled to room temperature to prevent reabsorption of moisture before use. At this stage, the eggshell powder is ready for advanced applications in research, where its uniform characteristics support reliable experimentation and data accuracy. ES powder predominantly consists of calcium carbonate (CaCO₃), making up about 88 % of its composition. This high calcium carbonate content gives eggshells their structural integrity, making them an excellent source of calcium for various applications [[21], [22], [23], [24]].

### Synthesis of ES/TiO2 composite photocatalyst

There are many ways to enhance the photocatalytic activity of TiO₂ by modifying its structure to degrade organic pollutants in water [[25], [26], [27], [28]]. The ES/TiO₂ composite photocatalyst is one such modification aimed at improving the photocatalytic performance of TiO₂. The ES/TiO_2_ composite photocatalyst was prepared using the solid-state dispersion (SSD) method in the ratios of 9:1, 8:2, and 7:3, the composite photocatalyst was ready and compared to ES and 100 % TiO^2^.

**Fig. 2:**
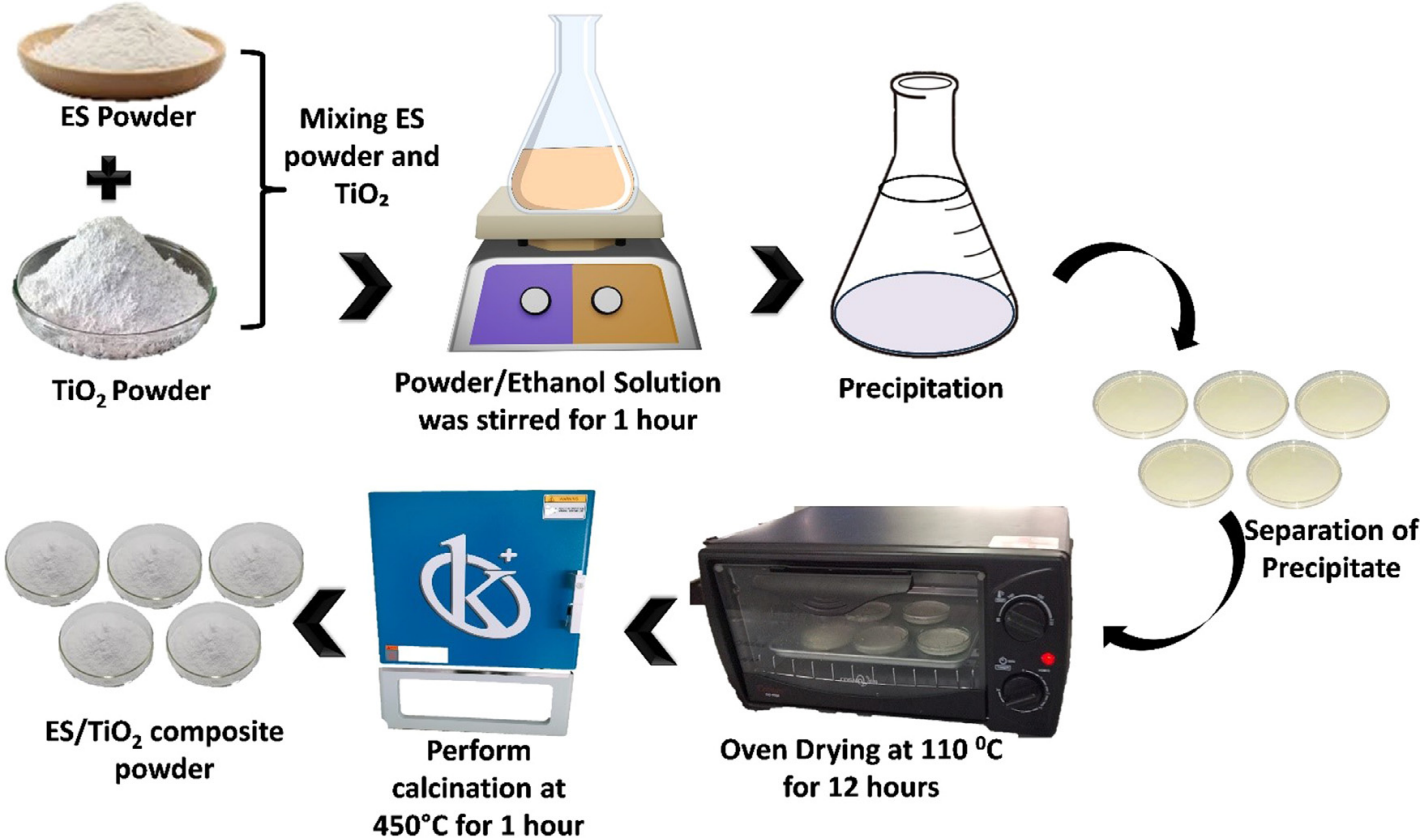
A schematic illustration of the ES/TiO₂ composite material synthesis process using Solid State Dispersion (SSD).

The synthesized eggshell powder (ES) was carefully weighed to 5 grams and placed in a sample container, while 5 grams of TiO₂ powder was prepared separately. For the synthesis of the ES/TiO₂ composite, the materials were mixed in varying mass ratios of 9:1, 8:2, and 7:3, with a total mixture mass of 5 grams. Specifically, the 9:1 ratio consisted of 4.5 grams of ES and 0.5 grams of TiO₂; the 8:2 ratio consisted of 4 grams of ES and 1 gram of TiO₂; and the 7:3 ratio consisted of 3.5 grams of ES and 1.5 grams of TiO₂. To ensure a homogeneous distribution of TiO₂ within the ES powder, the components were thoroughly blended using a spatula or stirrer. Subsequently, 100 mL of 95 % ethanol was added to each mixture and stirred with a magnetic stirrer at room temperature for 1 h. The resulting mixture was allowed to settle, after which the sediment was transferred to a petri dish and dried in an oven at 110 °C for 12 h. Finally, the dried mixture was calcined in a furnace at 450 °C for 1 h to produce the ES/TiO₂ composite material. The synthesized composite was then analyzed and tested for its performance [29,30]. A schematic illustration of the ES/TiO₂ composite material synthesis process is shown in Fig. 2.

### Photocatalytic activity measurement

**Fig. 3:**
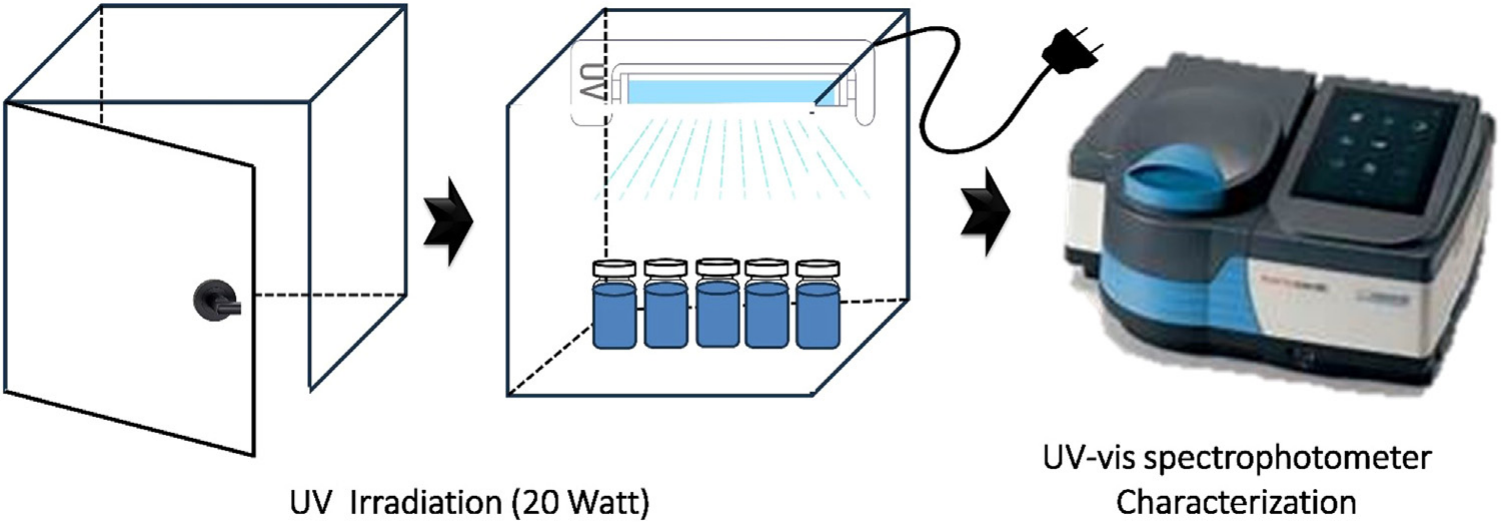
A schematic illustration of Photocatalytic activity measurement.

Methylene blue (MB) was used as a model pollutant in this study. Time-dependent degradation experiments were conducted to evaluate the photocatalytic activity of the prepared samples. A sample of 0.1 g was added to 20 mL of MB solution, and the mixture was irradiated with a 20-watt UV lamp (T8 20W, 60 cm, Evaco) at time intervals of 0 h, 4 h, 8 h, 12 h, 16 h, 20 h, and 24 h (Fig. 3).

The photocatalytic activity of the sample was determined by the degradation rate, calculated using Eq. (1):

(1) $$\%D=\frac{C_{0}-C_{t}}{C_{0}}x100\%$$

where *C_0_* and *C_t_* are the concentration of MB at the maximum absorption peak $\left(\lambda_{max}=665\;nm\right)$ before and after irradiation, respectively.

## Method validation

### ES/TiO2 composite synthesis results

The ES/TiO₂ composite was successfully synthesized using the Solid-State Dispersion method. The process yielded a homogeneous composite material, with TiO₂ effectively integrated onto the eggshell surface.

**Fig. 4:**
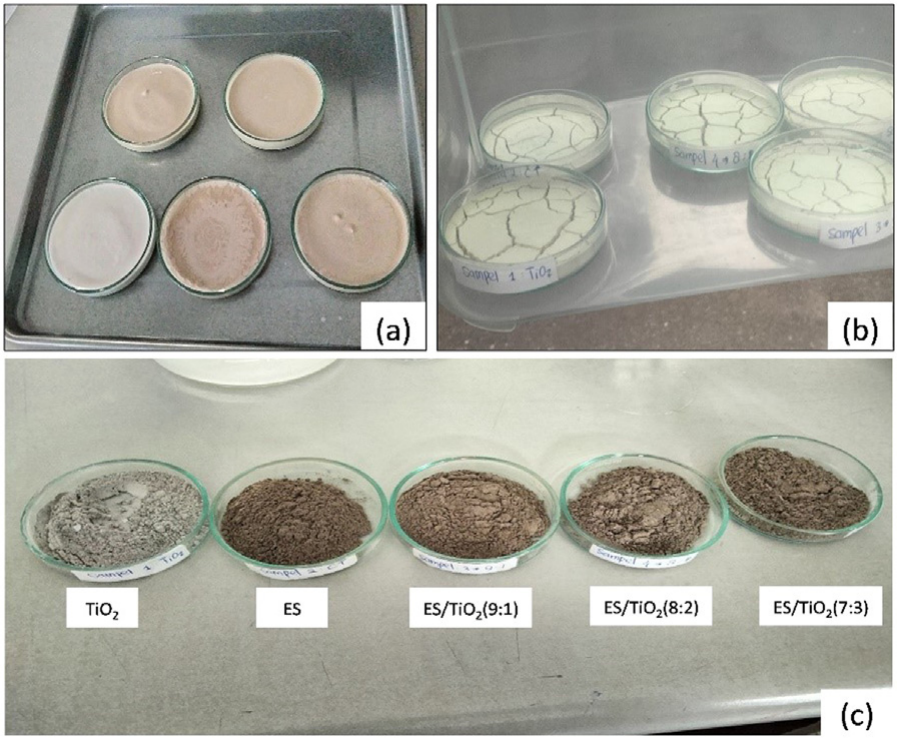
Drying process of samples, (a) Before oven drying, (b) After oven drying at 110 °C for 12 h and (c) Samples after calcination at 450 °C.

Fig. 4(a) Before oven drying. At this stage, the synthesized composite has not undergone significant drying. The sample still contains moisture, especially residual solvent (95 % ethanol) used during the mixing and dispersion process. This moisture needs to be removed to enhance the physical stability of the composite and prevent any potential negative effects on the material's photocatalytic activity. At this point, the sample typically has a damp texture, is softer, and may undergo physical or chemical changes during the drying process.

Fig 4(b) After oven drying at 110 °C for 12 h, the moisture content in the composite is significantly reduced, resulting in a drier and more stable material. At this temperature, any remaining solvent evaporates, and the composite becomes more homogeneous with a denser structure. This process strengthens the bonding between the ES (eggshell) and TiO₂ particles, ensuring a uniform distribution of the material on the eggshell surface. The result is a dry composite with a harder texture, ready for further steps such as photocatalytic testing or material characterization.

After oven drying and confirming that no solvent remained, the sample was subjected to calcination at 450 °C for 1 h. Following the calcination process, the resulting composite exhibited a gray color and had a fine powder texture as shown in Fig. 4(c). The composite was synthesized in three composition variations, with ES to TiO₂ ratios of 9:1, 8:2, and 7:3.

Visual observations indicate that the grayish-brown color observed in the composite is likely due to carbon residues formed from organic compounds in the eggshell (such as proteins and fats) that were not completely burned during the calcination process at 450 °C. At this temperature, most organic materials are expected to degrade; however, the combustion process may not be entirely efficient due to the relatively low calcination temperature compared to the temperatures above 600 °C that are typically required for complete combustion of organic materials.

TiO₂ powder obtained after the calcination process exhibits a fine, uniform texture with a grayish color. This indicates that the titanium dioxide has undergone successful thermal treatment, leading to the removal of organic impurities and moisture. The calcination process enhances the crystallinity of TiO₂, resulting in improved photocatalytic properties due to increased surface area and active sites for photocatalytic reactions. The powder's morphology typically appears as a smooth and homogeneous structure, ideal for applications in photocatalysis and other related fields (Fig. 4(c)).

ES Powder shows a slightly off-white or brownish-gray color due to the presence of residual carbon and other organic compounds that may not have fully decomposed during the calcination process. The texture of the ES powder is generally fine but can be coarser than that of TiO₂. The calcination process enhances the calcium carbonate (CaCO₃) content in the eggshell, converting it into calcium oxide (CaO), which can provide additional benefits in composite applications, such as enhancing adsorption properties and acting as a catalyst support. The color change of eggshells (ES) after calcination depends on the temperature and duration of the process. At temperatures below 800 °C, the resulting ES powder appears grayish-brown [6]. The morphology of the ES powder may also exhibit a more porous structure compared to TiO₂, which can be advantageous for specific applications, such as adsorbing pollutants in water purification (Fig. 4(c)).

The ES/TiO₂ composites with different ratios exhibit significant variations in the texture and morphology of the resulting powders:

1. ES/TiO₂ Ratio 9:1:This ratio produces a softer powder with slightly larger particles. This is attributed to the dominance of the eggshell (ES) material, which does not completely decompose during the synthesis process. The higher ES content results in residues that are not perfectly dispersed; however, this composite demonstrates stronger adsorption characteristics.
2. ES/TiO₂ Ratio 8:2:This composition yields a more homogeneous texture with better particle distribution between ES and TiO₂. The particles in this composite are evenly spread, resulting in a fine and uniform powder. This ratio reflects an ideal balance between the adsorptive properties of ES and the photocatalytic activity of TiO₂, making it one of the most efficient composites.
3. ES/TiO₂ Ratio 7:3:This ratio produces the finest powder, showing effective dispersion of TiO₂ particles. Nonetheless, the grayish hue of this powder is still noticeable, likely due to the presence of carbon residues from the eggshell that have not fully decomposed during the calcination process.

Additionally, this color change may also be associated with the interaction between the CaCO₃ component in the eggshell and TiO₂ during calcination, which could lead to slight modifications in the surface properties of the material, resulting in a grayish-brown hue. This color is distinct from the bright white typically produced from the calcination of pure TiO₂, indicating the influence of the ES material on the final composite outcome.

The fine powder texture of the composite indicates that the grinding and calcination processes successfully reduced the particle size. This fine powder is ideal for water purification applications, as the smooth texture allows for an increased surface area, which is crucial for enhancing adsorption effectiveness and photocatalytic reactions. Composites with a higher ES ratio (9:1) tend to be lighter and slightly coarser compared to those with a higher TiO₂ ratio (7:3), which feel denser and smoother due to the increased TiO₂ content.

## UV–Vis analysis

The ultraviolet and visible region absorption analysis was observed by using UV–Vis spectroscopy THERMO SCIENTIFIC ORION AquaMate with range 665–700 nm. Photocatalytic activity of all composites was studied by evaluating the rate of MB degradation under irradiation by UV Lamp 20W.

**Fig. 5:**
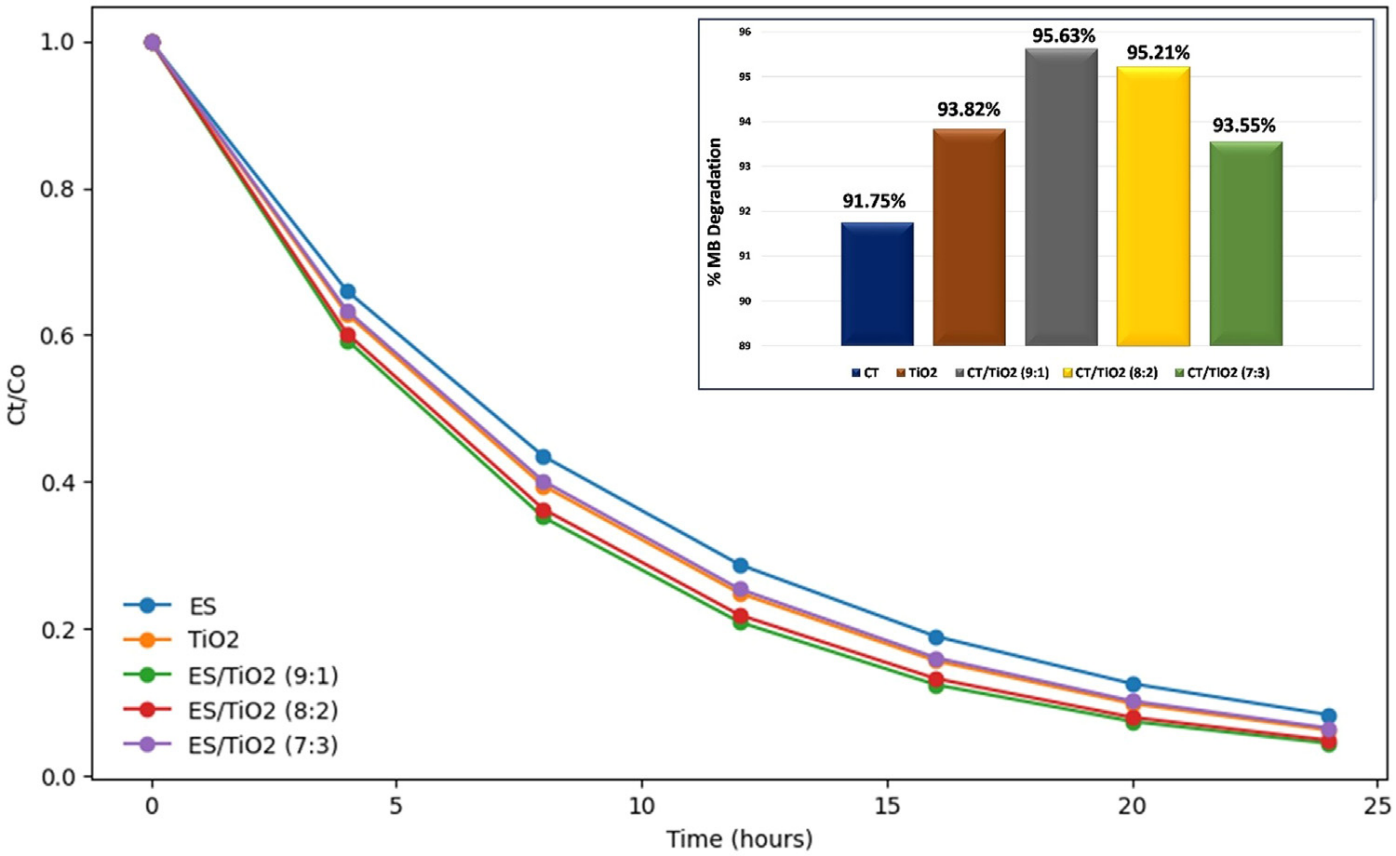
(a) Normalized degradation behavior of MB using different types of photocatalyst and (b) MB percentage degradation.

Fig. 5 depicts a significant decrease in the absorbance of Methylene Blue (MB) over time. This decrease follows an exponential decay pattern, indicating that the degradation of MB follows first-order reaction kinetics. This suggests that the rate of MB degradation is directly proportional to the concentration of MB remaining at any given time. Fig. 5(a) illustrates the normalized degradation behavior of MB using different photocatalysts. From this graph, it is evident that each photocatalyst exhibits a distinct pattern of MB concentration reduction. The ES/TiO₂ (9:1) composition appears to be the most effective, with a faster concentration reduction compared to other photocatalysts.

The percentage degradation of MB is calculated using Eq. (1) with *C_0_ = 1.442 PPM*, for samples irradiated for 24 h (Fig. 5(b)). A higher degradation percentage indicates the material's effectiveness in degrading MB. From the degradation plot, it is observed that the CT/TiO₂ (9:1) composition demonstrates the highest MB degradation level, with a value of 95.63 %. The higher effectiveness of ES/TiO₂ (9:1) is likely due to the combination of the adsorption capacity of the eggshell and the photocatalytic activity of TiO₂.

As the irradiation time increases, the reduction in MB absorbance becomes more pronounced, indicating that the hydroxyl radicals generated on the surface of the photocatalyst increase over time. This accelerates the MB degradation process, optimizing the photocatalytic effectiveness with longer exposure.

## Photocatalytic mechanism

**Fig. 6:**
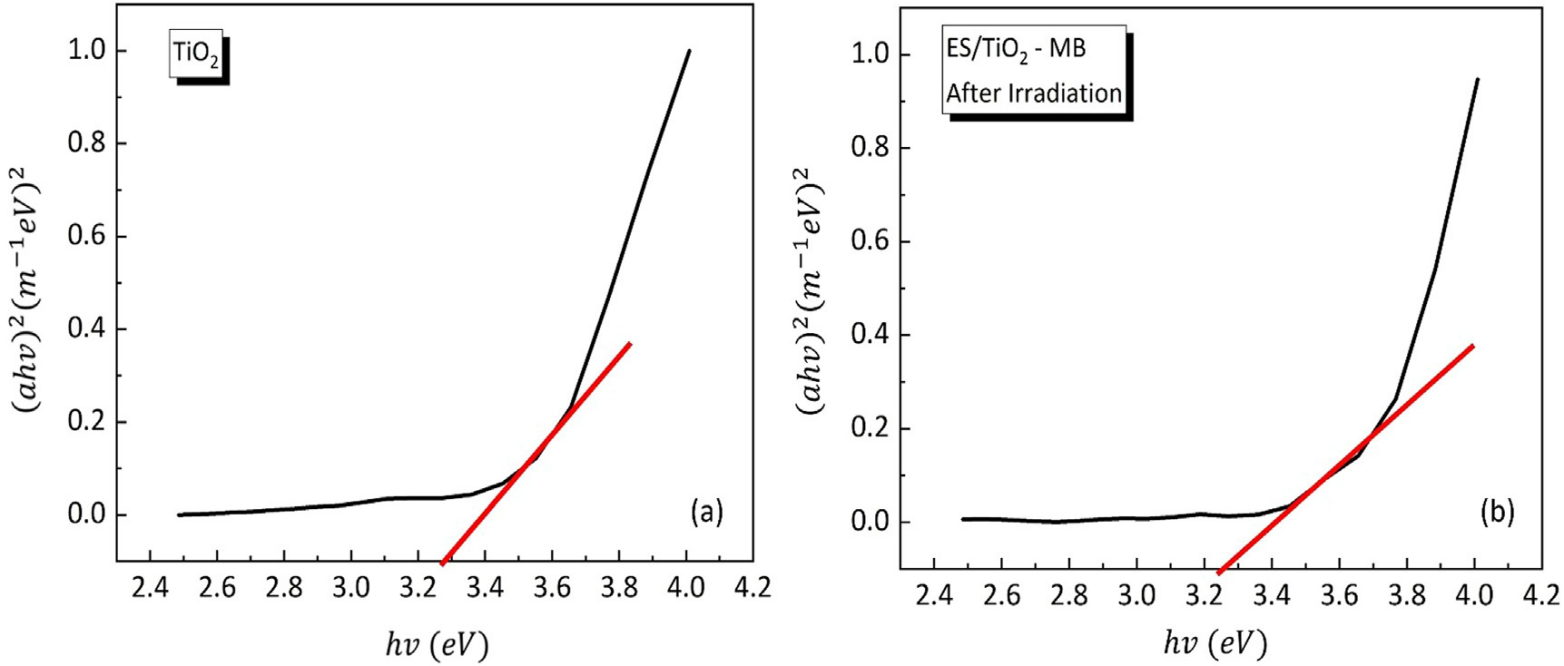
Graph of band gap calculation using the Tauc Plot method: (a) band gap of 3.27 eV for TiO₂ and (b) band gap of 3.25 eV for ES/TiO₂.

Based on the band gap calculations using the Tauc Plot method, the pure TiO₂ photocatalyst exhibited a band gap of 3.27 eV, which decreased to 3.25 eV after compositing with eggshell (ES), as shown in Fig. 6. This slight reduction indicates an enhancement in photocatalytic performance, which can be attributed to the synergistic effect between TiO₂ and the structural support provided by CaCO₃ in the eggshell. The band gap value of 3.27 eV for TiO₂ was also reported in previous research [31]. Furthermore, the band gap of the ES/TiO₂ photocatalyst in solution was found to be 2.45 eV, reflecting its potential for visible light absorption and its significant role in accelerating the degradation of methylene blue. After 24 h of UV exposure, the methylene blue solution treated with ES/TiO₂ showed an increase in band gap to 3.25 eV, accompanied by a shift towards a more colorless solution. This behavior highlights the effectiveness of the composite in breaking down organic dye pollutants. The observed band gap values are consistent with reported ranges of 2.27–5.54 eV [32], reinforcing the potential of ES/TiO₂ composites for photocatalytic applications, especially those requiring precise tuning of band gap properties.

Eggshell improves the photocatalytic performance of TiO₂ composites due to the presence of CaCO₃, which acts as a structural support and enhances the dispersion of TiO₂ particles. Previous studies have shown that CaCO₃-loaded TiO₂ composites exhibit uniform and complete loading of TiO₂ nanoparticles. The dispersibility of TiO₂ is significantly enhanced compared to pure TiO₂, leading to improved photocatalytic efficiency. This improvement is attributed to the strong chemical bonds formed between the CaCO₃ and TiO₂ particle interfaces, which facilitate better interaction and more efficient electron transfer during the photocatalytic process. These mechanisms contribute to the enhanced photocatalytic performance of the CaCO₃–TiO₂ composite, reducing the amount of TiO₂ required while maintaining high efficiency [33].

The COD (Chemical Oxygen Demand) test results showed that the oxygen demand of the methylene blue solution to oxidize inorganic materials was 32 mgO₂/L. After applying ES/TiO₂ without UV irradiation, the COD value increased to 24 mgO₂/L. This indicates that the photocatalyst molecules were evenly distributed within the sample, raising the COD value due to TiO₂ detected in the sample. However, after 24 h of UV irradiation, the COD value of the methylene blue sample significantly decreased to 8 mgO₂/L. This suggests that the ES particles reduced the TiO₂ composition in the sample solution, thereby lowering the chemical content, which demonstrates the effectiveness of ES/TiO₂ as a photocatalyst in reducing dyes and chemicals in methylene blue solutions. The results were below the wastewater quality standard, which means it is safe to be processed into drinking water for the community based on the Regulation of The Minister of Environment of The Republic of Indonesia Number 5 Year 2014 Concerning Wastewater Quality Standards Concerning Wastewater Quality Standards for Textile Industry Businesses and/or Activities.

## XRD analysis

**Fig. 7:**
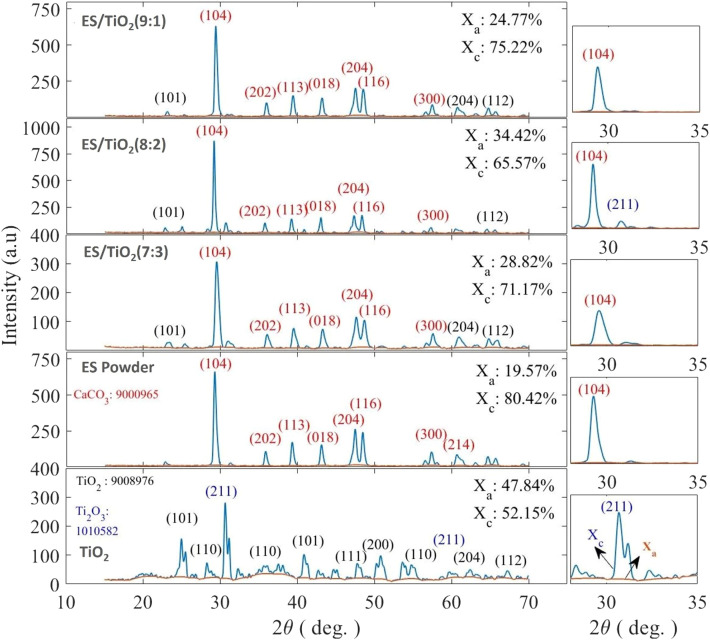
XRD characterization results for the samples: TiO₂, eggshell (ES), and ES/TiO₂ composites with composition variations of 9:1, 8:2, and 7:3.

The XRD characterization results for the samples: TiO₂, eggshell (ES), and three variations of the ES/TiO₂ composites (9:1, 8:2, 7:3) are shown in Fig. 7.

TiO₂ exhibits both Anatase phase (211) and Rutile phase (110, 101, 111). The dominant peak appears at around 2ϴ = 25°, indicating the presence of TiO₂ in the anatase form [34]. Additionally, there are extra peaks corresponding to the rutile phase. The ratio of Anatase to Rutile phase: XRD analysis shows that the pure TiO₂ sample contains a ratio of anatase phase (47.84 %) to rutile phase (52.15 %). This indicates that the sample contains a mixture of phases, with rutile being the dominant phase. Characteristic peaks of CaCO₃ (104, 113, 018): The eggshell sample displays a dominant peak at approximately 29.5°, indicating CaCO₃ (calcite) as the main phase. Phase Composition: The eggshell (ES) primarily consists of calcite (CaCO₃), with a calcite phase percentage of 80.42 %.

TiO₂ exhibits both Anatase phase (211) and Rutile phase (110, 101, 111). The dominant peak appears at around 2ϴ = 25°, indicating the presence of TiO₂ in the anatase form. Additionally, there are extra peaks corresponding to the rutile phase. The ratio of Anatase to Rutile phase: XRD analysis shows that the pure TiO₂ sample contains a ratio of anatase phase (47.84 %) to rutile phase (52.15 %). This indicates that the sample contains a mixture of phases, with rutile being the dominant phase. Characteristic peaks of CaCO₃ (104, 113, 018): The eggshell sample displays a dominant peak at approximately 29.5°, indicating CaCO₃ (calcite) as the main phase. Phase Composition: The eggshell (ES) primarily consists of calcite (CaCO₃), with a calcite phase percentage of 80.42 %. Phase TiO₂ and CaCO₃ (Calcite): The three ES/TiO₂ composite samples exhibit peaks from two main phases, namely anatase TiO₂ and CaCO₃ (calcite) from the ES. The variation of the Anatase ratio (Xₐ) and Calcite (Xₐ) is presented in the figure with ES/TiO₂ (9:1): Anatase phase 24.77 %, Calcite 75.22 %. This indicates that the proportion of TiO₂ is lower compared to calcite. ES/TiO₂ (8:2): Anatase phase 34.42 %, Calcite 65.57 %. The anatase ratio is slightly higher compared to ES/TiO₂ (7:3), but calcite remains dominant. ES/TiO₂ (7:3): Anatase phase 28.82 %, Calcite 71.17 %. The proportion of anatase falls between the 9:1 and 8:2 compositions, indicating that the TiO₂ phase is still significant but not yet dominant.

The XRD data for TiO₂ shows a mixture of anatase and rutile phases, with rutile being slightly more dominant. The anatase phase is typically more photocatalytically active. The presence of CaCO₃ in the composite, indicated by the strong CaCO₃ peaks, suggests that the eggshell contributes significantly to the composite structure, particularly in the calcite phase, which serves as a mechanical support material. The TiO₂ content in the composite exhibits a lower anatase ratio, indicating that calcite from the eggshell remains the dominant phase.

## HRTEM observation

**Fig. 8:**
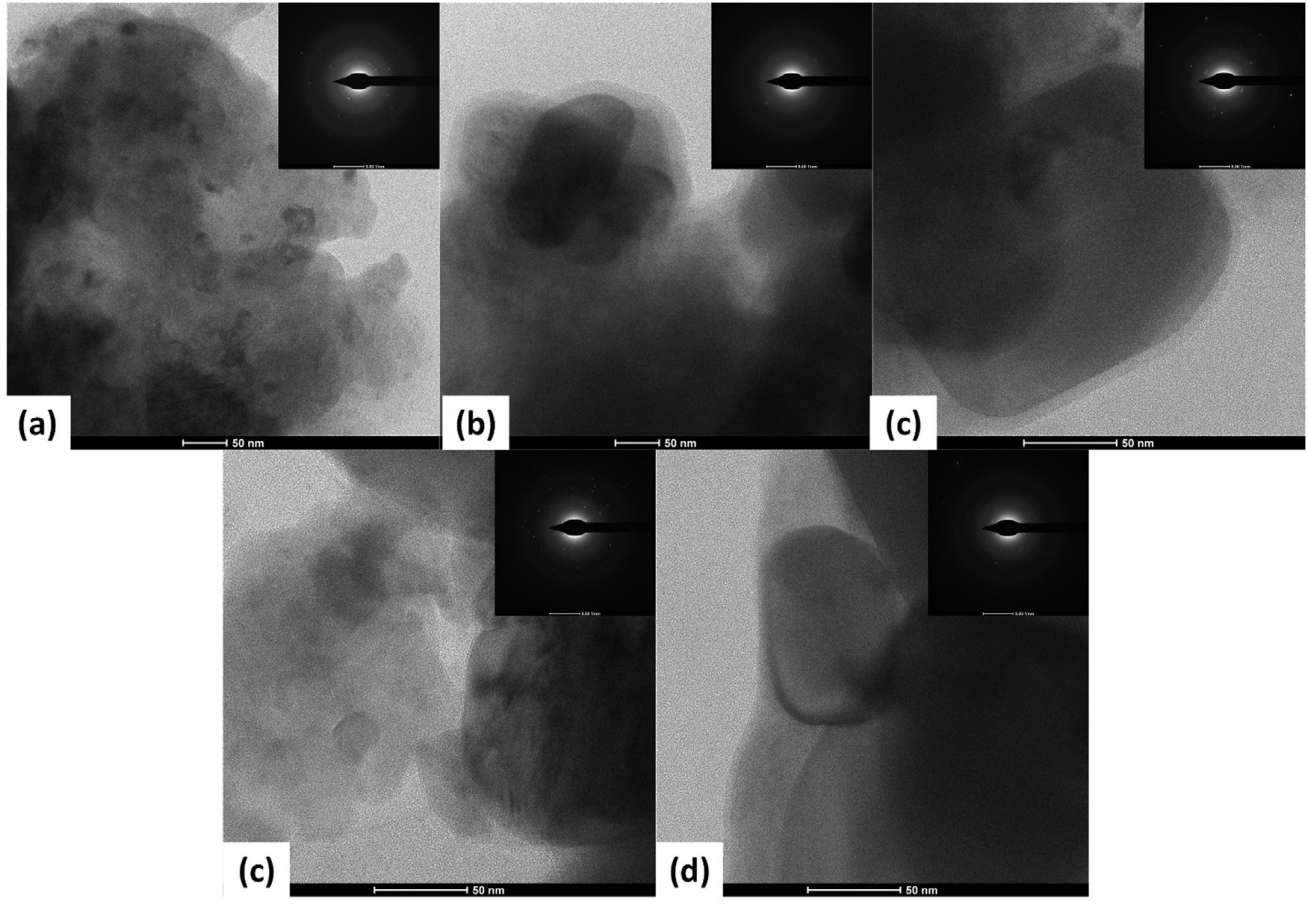
HRTEM images of (a) eggshell powder (ES), (b) TiO₂, (c) ES/TiO₂ composite with a 9:1 ratio, (d) ES/TiO₂ composite with an 8:2 ratio, and (e) ES/TiO₂ composite with a 7:3 ratio. Each image presents the morphological structure and the Selected Area Electron Diffraction (SAED) patterns of the respective samples.

High-resolution transmission electron microscopy (HRTEM) TALOS F200C G2 was used to get more detailed information about the morphology and structure of the composite. The High-Resolution Transmission Electron Microscopy (HRTEM) images (Fig. 8 illustrate the morphological characteristics of eggshell powder (ES), TiO₂, and ES/TiO₂ composites with varying ratios (9:1, 8:2, and 7:3). The ES powder (Fig. 8a) reveals large, irregular particles with a rough surface, and its Selected Area Electron Diffraction (SAED) pattern shows weak diffraction rings, suggesting an amorphous or low-crystallinity structure. This amorphous nature likely limits its photocatalytic activity when used alone. In contrast, TiO₂ (Fig. 8b) exhibits smaller particles and a more ordered morphology, with distinct SAED diffraction rings indicating higher crystallinity, likely in the anatase or rutile phase, which enhances its photocatalytic efficiency.

For the ES/TiO₂ composite with a 9:1 ratio (Fig. 8c), the morphology shows larger ES particles integrated with finer TiO₂ structures, while the SAED pattern still displays TiO₂ diffraction rings, though with reduced intensity, implying an interaction between ES and TiO₂ that slightly affects crystallinity. This structural combination improves photocatalytic performance, as the ES supports TiO₂, enhancing light absorption and aiding in methylene blue (MB) degradation. The 8:2 composite (Fig. 7d) shows a more homogeneous particle distribution with better integration between ES and TiO₂, and a stronger SAED diffraction intensity, indicating higher crystallinity. However, the increased TiO₂ content may alter the distribution of active surface areas and light absorption, potentially lowering the efficiency of MB degradation compared to the 9:1 ratio.

Finally, the 7:3 composite (Fig. 8e) displays an even distribution of particles and a denser structure, with a well-defined SAED pattern reflecting increased crystallinity due to the higher TiO₂ concentration. However, this higher concentration may lead to aggregation, reducing the active surface area and potentially hindering MB degradation efficiency.

Based on the UV–Vis results indicating that the ES/TiO₂ (9:1) composition is most effective in degrading Methylene Blue (MB), this effectiveness can be linked to several factors observed in the HRTEM analysis. First, the 9:1 ratio results in a more uniform distribution of TiO₂ particles and sufficiently small particle size to maximize the active surface area. This allows more photons from light to be absorbed by TiO₂, which serves as the primary photocatalyst.

The 9:1 ratio provides a more uniform distribution of TiO₂ particles in the composite, which minimizes agglomeration and increases the active surface area, thereby enhancing the efficiency in the degradation process of organic pollutants such as methylene blue. Based on the HRTEM results, the TiO₂ particles at this ratio are well-dispersed, preventing agglomeration and ensuring a more effective photocatalytic reaction (Fig. 8c). The combination of ES and TiO₂ at this ratio facilitates optimal contact between TiO₂ surfaces and MB molecules, enhancing the degradation process. The degradation efficiency for the 9:1 composition is 95.63 %, outperforming the 8:2 (95.21 %) and 7:3 (93.55 %) compositions. This highlights the superior photocatalytic performance at the 9:1 ratio. The nano-sized particles at the 9:1 ratio may also exhibit a synergistic interaction between TiO₂ and components within the ES, contributing to improved photocatalytic efficiency. At higher ratios of TiO₂, such as 8:2 or 7:3, a decrease in photocatalytic activity may occur due to TiO₂ particle agglomeration, which reduces the effective surface area.

## Conclusion

The ES/TiO₂ composite photocatalyst was successfully prepared using the Solid-State Dispersion (SSD) method in various ratios. Adsorption and photocatalytic activity tests showed that the ES/TiO₂ composite with 10 wt% TiO₂ and 90 wt% eggshell exhibited the highest MB degradation within 24 h under UV irradiation in suspension conditions, outperforming other composite ratios. As a composite photocatalyst, eggshell shows high adsorption potential despite its micro-sized nature and could be even more effective in nanoscale form. With its high adsorption capacity, eggshell serves as a valuable material for removing reactive dyes like MB. This supports sustainability efforts by reusing eggshell waste in an eco-friendly manner, and the composite holds significant potential for wastewater treatment applications.

## Limitations

Not applicable.

## Ethics statements

Not applicable.

## CRediT authorship contribution statement

**Rahmawati Munir:** Conceptualization, Supervision, Writing – original draft. **Dadan Hamdani:** Investigation, Data curation, Methodology. **Darnah:** Data curation, Formal analysis, Writing – review & editing. **Eva Marliana:** Data curation, Writing – review & editing. **Rahmiati Munir:** Writing – review & editing. **Sahara Hamas Intifadhah:** Writing – original draft, Writing – review & editing. **Ratna Kusuma:** Writing – review & editing.

## Declaration of competing interest

The authors declare that they have no known competing financial interests or personal relationships that could have appeared to influence the work reported in this paper.

## Data Availability

Data will be made available on request.
